# Hemodynamic Impact of the Aberrant Subclavian Artery: A CFD Investigation

**DOI:** 10.3390/jpm15120603

**Published:** 2025-12-05

**Authors:** Edoardo Ugolini, Giorgio La Civita, Marco Ferraresi, Moad Alaidroos, Alessandro Carlo Luigi Molinari, Maria Katsarou, Giovanni Rossi, Emanuele Ghedini

**Affiliations:** 1 Industrial Engineering Department, University of Bologna, 40126 Bologna, Italy; emanuele.ghedini@unibo.it; 2 Independent Researcher, 40127 Bologna, Italy; giorgio.lacivita.ing@gmail.com; 3 Division of Vascular Surgery, Alessandro Manzoni Hospital, 23900 Lecco, Italy; m.alaidroos@asst-lecco.it (M.A.); a.molinari@asst-lecco.it (A.C.L.M.); m.katsarou@asst-lecco.it (M.K.); g.rossi@asst-lecco.it (G.R.)

**Keywords:** thoracic aorta, aberrant artery, CFD, thoracic aneurysm

## Abstract

**Background/Objectives**: The aberrant subclavian artery (ASA) represents the most common congenital anomaly of the aortic arch, and is frequently associated with a Kommerell diverticulum, an aneurysmal dilation at the anomalous vessel origin. This condition carries a significant risk of rupture and dissection, and growing evidence indicates that local hemodynamic alterations may contribute to its development and progression. Computational Fluid Dynamics (CFD) provides a valuable non-invasive modality to assess biomechanical stresses and elucidate the pathophysiological mechanisms underlying these vascular abnormalities. **Methods**: In this study, twelve thoracic CT angiography scans were analyzed: six from patients with ASA and six from individuals with normal aortic anatomy. CFD simulations were performed using OpenFOAM, with standardized boundary conditions applied across all cases to isolate the influence of anatomical differences in flow behavior. Four key hemodynamic metrics were evaluated—Wall Shear Stress (WSS), Oscillatory Shear Index (OSI), Drag Forces (DF), and Turbulent Viscosity Ratio (TVR). The aortic arch was subdivided into Ishimaru zones 0–3, with an adapted definition accounting for ASA anatomy. For each region, time- and space-averaged quantities were computed to characterize mean values and oscillatory behavior. **Conclusions**: The findings demonstrate that patients with ASA exhibit markedly altered hemodynamics in zones 1–3 compared to controls, with consistently elevated WSS, OSI, DF, and TVR. The most pronounced abnormalities occurred in zones 2–3 near the origin of the aberrant vessel, where disturbed flow patterns and off-axis mechanical forces were observed. These features may promote chronic wall stress, endothelial dysfunction, and localized aneurysmal degeneration. Notably, two patients (M1 and M6) displayed particularly elevated drag forces and TVR in the distal arch, correlating with the presence of a distal aneurysm and right-sided arch configuration, respectively. Overall, this work supports the hypothesis that aberrant hemodynamics contribute to Kommerell diverticulum formation and progression, and highlights the CFD’s feasibility for clarifying disease mechanisms, characterizing flow patterns, and informing endovascular planning by identifying hemodynamically favorable landing zones.

## 1. Introduction

The ASA (aberrant subclavian artery) in a left-sided aortic arch is the most frequent aortic arch anatomical anomaly, occurring in 0.5–1% of the population [1]. A rarer variant of this congenital anomaly is the presence of an ALSA (aberrant left subclavian artery). In the setting of a right-sided aortic arch, this combination occurs in 0.04–0.4% of the general population [2]. In patients with ARSA (aberrant right subclavian artery), the aortic arch typically gives rise to four supra-aortic branches, in the following order: the right common carotid artery, the left common carotid artery, the left subclavian artery, and finally the ARSA. The anatomy is specular in patients with ALSA. In certain cases, a bicarotid trunk may be present, from which both common carotid arteries originate. This condition is a developmental error in which the right dorsal aorta involutes proximally to the right subclavian artery, leaving it attached to the descending thoracic aorta. Aneurysmal degeneration of the ASA ostium, defined as a diameter more than 1.5 times the diameter of the distal ASA, is reported in 20 to 60% of cases and is known as KD (Kommerell Diverticulum) [3,4]. Although KD is usually asymptomatic in adults, it carries a 19% to 53% risk of rupture or dissection with life-threatening implications [5].

This elevated risk of aneurysmal degeneration, rupture, and dissection has been explained by many authors through histological findings showing a high prevalence of cystic medial necrosis within the wall of resected KD [6,7]. Nonetheless, it has also been hypothesized that altered hemodynamics associated with this anomalous anatomy—resulting in increased WSS (Wall Shear Stress)—may play a significant role in explaining the elevated risk of complications involving the KD [8]. Supporting the latter hypothesis, numerous lines of evidence have emerged in recent years suggesting that abnormal flow patterns associated with altered vascular geometry may contribute to endothelial dysfunction and inflammation, aneurysmal degeneration, intimal thickening, and atherosclerotic plaque formation [9,10,11]. In this research context, CFD (Computational Fluid Dynamics) has become an important non-invasive tool for investigating and quantifying the biomechanical forces acting on the vessel wall and within the blood flow itself.

With specific reference to TEVAR (Thoracic Endo Vascular Aortic Repair), CFD has assumed a central role across multiple areas, including: the elucidation of disease pathogenesis; preoperative planning—especially in assessing drag forces (DF) acting on the proximal landing zone to mitigate risks of device migration and endoleak; and the construction of predictive models for postoperative complications by simulating hemodynamic conditions and stent–vessel interactions [12,13].

The aim of this study is to preliminarily investigate the role of hemodynamic factors, analyzed through CFD, both in the development of aneurysmal degeneration of the KD and its high risk of rupture and dissection, as well as in potential surgical planning, particularly in terms of selecting the appropriate proximal landing zone for TEVAR. This study is intended as a feasibility investigation for the use of CFD for the diagnosis of this rare congenital anomaly, bearing in mind that there is a general lack of studies that investigate this pathology from a fluid dynamic point of view. By gathering all data harvested during the CFD campaign in an open and growing repository, this feasibility study is presented as a starting point from which to understand and characterize such a rare anatomical anomaly, by means of more complete works on a larger patient spectrum.

## 2. Materials and Methods

This study employs CFD to investigate the hemodynamic implications of aortic arch anatomical variants characterized by the presence of an aberrant subclavian artery. The objective is to assess how morphological differences influence key fluid dynamic metrics, specifically: WSS, OSI (Oscillatory Shear Index), DF (Drag Force), and TVR (Turbulent Viscosity Ratio). These parameters are analyzed within the anatomical regions defined by the Ishimaru classification. A total of twelve patients were investigated, split equally into six healthy and six aberrant anatomies. All the patients who participated in this trial volunteered and provided written informed consent for this study. Patients with ASA anomalies who were treated or evaluated for treatment at our center in the last 5 years were retrospectively enrolled. For each of them, a patient with normal aortic arch anatomy matched for age and arch curvature was also enrolled.

The preoperative CTA (Computed Angiography Tomography) scans of patients with ASA and those of healthy subjects with normal anatomy were sent by the physicians to the engineers for aortic arch segmentation and subsequent CFD analysis. Surface reconstruction of the aortic lumen was obtained using ITK-Snap v 4.0.2. [14], a widely known active contour segmentation software in the biomedical engineering community. Aortic arches with normal anatomy were divided into four zones according to Ishimaru’s standard classification, whereas arches with ASA were divided into four zones according to Tinelli’s modified version of the classification [15]. CFD simulations were conducted using OpenFOAM v.2206 (The OpenFOAM Foundation), an open-source platform, where the standard PIMPLE solver is used, along with custom functions to compute the hemodynamic parameters explained later in the manuscript.

Post-processing of the CFD results focused on evaluating the spatial and temporal distribution of WSS, OSI, DF, and TVR, and comparing these hemodynamic quantities across different arch zones in the same patient and in the same zone in patients with normal and aberrant anatomy the regions specified by the Ishimaru classification, thereby highlighting the influence of anatomical variation on local and global flow dynamics. To account for any initial transient phenomena, three cardiac cycles have been simulated, and results are computed solely for the last one, to exclude numerical noise because of simulation initialization.

### 2.1. CFD Analysis Method

#### 2.1.1. Governing Equations

The Navier–Stokes system of equations is reported below. We considered transient, incompressible, isothermal, non-Newtonian, and turbulent flow conditions. A FSI (Fluid–Structure Interaction) approach for such pathology is not considered in this study because of the lack of reliability of the aortic wall’s mechanical properties. However, the general feasibility of the FSI modeling will be assessed in future studies. In its RANS (Reynolds Averaged Navier–Stokes) formulation, such a system can be written in its vectorial form as follows:

(1) ∇·u=0

(2) $$\rho \left(\frac{\partial u}{\partial t}+\left(u\cdot \nabla \right)u\right)+\nabla p-\nabla \cdot \left(\left(\mu +\mu_{t}\nabla u\right)\right)=\rho g$$

where u=u(x,t) is the velocity vector, p=p(x,t) the pressure field, ρ the density, which is assumed to be constant with a value of 1030 $\frac{kg}{m^{3}}$, μ=μ(x,t) the dynamic viscosity, $\mu_{t}$ turbulent dynamic viscosity, and g the gravitational acceleration.

For turbulent behavior, a k−ω SST turbulence model was used to resolve the turbulent flow features, offering a good compromise between near-wall accuracy and robustness in free-flow regions [16]. Here are reported the transport equations for k and ω:

(3) $$\frac{\partial \left(\rho k\right)}{\partial t}+\frac{\partial \left(\rho k\;u_{j}\right)}{\partial x_{j}}=P_{k}-\beta \rho \;k\;\omega +\frac{\partial }{\partial x_{j}}[\left(\mu +\sigma_{k}\;\mu_{t}\right)\frac{\partial k}{\partial x_{j}}]$$

(4) $$\frac{\partial \left(\rho \omega \right)}{\partial t}+\frac{\partial \left(\rho \omega \;u_{j}\right)}{\partial x_{j}}=\alpha (\frac{\omega }{k})P_{k}-\beta \rho \omega^{2}+\frac{\partial }{\partial x_{j}}[\left(\mu +\sigma_{\omega }\;\mu_{t}\right)\frac{\partial \omega }{\partial x_{j}}]+2\left(1-F^{1}\right)\rho \sigma_{\omega_{2}}\frac{1}{\omega }\frac{\partial k}{\partial x_{j}}\frac{\partial \omega }{\partial x_{j}}$$

A rigid wall assumption was adopted for all simulations, and wall compliance was therefore neglected. Blood was modeled as a non-Newtonian fluid using the Bird–Carreau model, which captures its shear-thinning behavior. Under the Bird–Carreau formulation, cinematic viscosity can be expressed as in the equation below, a function of the shear rate $\dot{\gamma }$:

(5) $$\mu =\mu_{\infty }+\frac{\mu_{0}-\mu_{\infty }}{{\left(1+{\left(\lambda \dot{\gamma }\right)}^{2}\right)}^{\frac{1-n}{2}}}$$

With $\mu_{0}=0.0001536\;Pa\cdot s$, $\mu_{\infty }=0.000003362\;Pa\cdot s$, n=0.2128, λ=8.2 s respectively the low-shear viscosity, high shear viscosity, power law constant, and time constant of the model.

#### 2.1.2. Numerical Methods

Governing equations are discretized and solved using a widely adopted set of solvers and schemes. Temporal discretization is performed using the Crank-Nicholson method with a blending factor equal to 0.5. Divergence is discretized using bounded Gauss linear upwind scheme, gradients are computed using cell limited Gauss linear scheme. Laplacian is discretized using Gauss linear limited and the interpolation scheme is linear. Wall and near wall zones treatment are handled by the meshwave method.

The set of linear system obtained by the numerical schemes is solved using the following numerical methods: Pressure Coupled Gradient for pressure with Simplified Diagonal-based Incomplete Cholesky as preconditioner for pressure, Preconditioned Bi-conjugate Gradient Stabilized solver for velocity and all turbulent quantities.

Further information on the discretization schemes and numerical methods can be found at the OpenFOAM documentation (https://www.openfoam.com/documentation/overview, accessed on 7 October 2025).

#### 2.1.3. Mesh Generation

**Figure 1:**
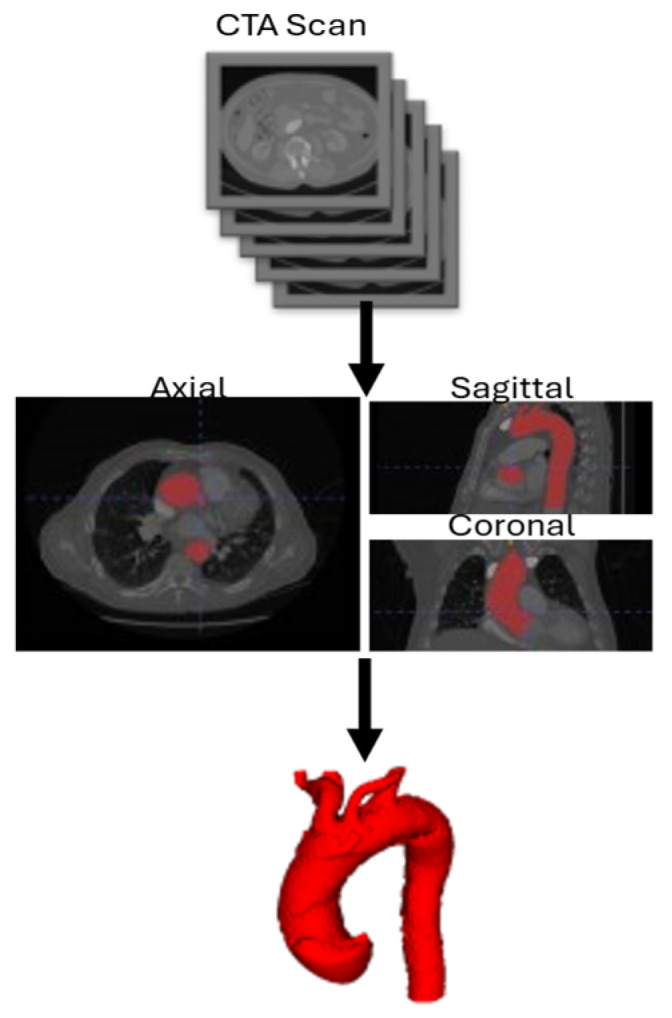
3D surface reconstruction workflow. CTA: Computed Tomography Angiography.

The computational geometry was reconstructed from CTA scans. CTA scans used in this study are characterized by Slice Thickness (ST) of 0.625 mm or 1 mm, Pixel Spacing of 0.751973 mm and Iomeron-400 as contrast medium. A semi-automatic segmentation process was performed using ITK-Snap, followed by surface mesh cleaning and smoothing before volume meshing. The domain includes the aortic root, the supra-aortic vessels, and the descending aorta up to the level of the celiac trunk. Once the CTA Scan DICOM (Digital Imaging and Communications in Medicine) series was obtained, the aortic lumen was isolated using upper and lower threshold values, generating a binary image. Finally, after placing growth bubbles inside the interest area, the aortic lumen was segmented following an active contouring algorithm. Single inlet, outlet, and wall patches are extracted from the geometry, obtaining the final merged surface to provide to the mesh generator. Aortic arch zones were defined by creating a plane perpendicular to the centerline passing through the most distal portion of the ostium of the supra-aortic trunk included in that specific zone. The mesh generation process is schematized in Figure 1. The mesh generator adopted in this study is cartesianMesh, native in the OpenFOAM environment. Boundary layer creation has been implemented to capture near-wall phenomena and properly solve the governing equations. The mesh consisted, depending on the complexity of the anatomy, of 55,000 to 80,000 tetrahedral elements. For each of the twelve acquisitions, the same medical image protocol, image processing, and surface mesh reconstruction were used.

#### 2.1.4. Boundary Conditions

Boundary conditions were derived from a synthetic dataset generated by a 1D cardiovascular model [17]. At the aortic root (inlet) and supra-aortic branches (outlets), time-varying velocity profiles were imposed along with zero-gradient pressure conditions.

At the descending aorta outlet, a time-varying reduced pressure profile was applied, coupled with a zero-gradient velocity condition. Here are reported the boundary conditions in their mathematical formulation:

Inlet – Aortic Root

$$u\left(x,t\right)=u_{inlet}\left(t\right),\forall x\in \Gamma_{in},\forall t\in [0,T]$$

$$\nabla p\cdot n=0,\forall x\in \Gamma_{in},\forall t\in [0,T]$$

Outlet – Supra-Aortic Vessels

$$u\left(x,y\right)=u_{out}^{ref}\left(t\right),\forall x\in \Gamma_{out,i}\;i=1,\ldots ,N\;,\forall t\in \left[0,T\right]$$

$$\nabla p\cdot n=0,\forall x\in \Gamma_{out,i}$$

Outlet – Descendant Aorta

$$p\left(x,t\right)=p_{desc}\left(t\right),\forall x\in \Gamma_{desc},\forall t\in \left[0,T\right]$$

$$\nabla u\cdot n=0,\forall x\in \Gamma_{desc},\forall t\in [0,T]$$

Wall

$$u\left(x,t\right)=0,\forall x\in \Gamma_{wall},\forall t\in \left[0,T\right]$$

**Figure 2:**
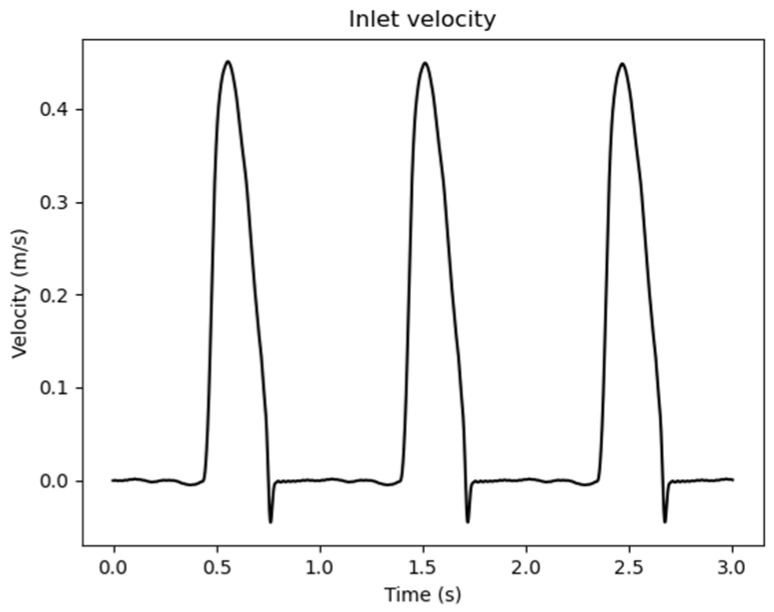
Inlet waveform velocity values adopted as boundary conditions.

**Figure 3:**
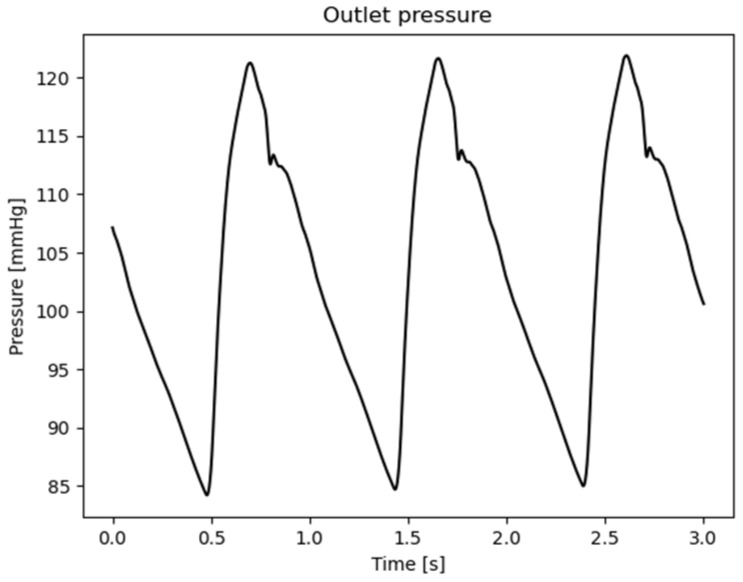
Outlet waveform pressure values adopted as boundary conditions.

With $\Gamma_{in}$, $\Gamma_{out,i}$, $\Gamma_{desc}$, $\Gamma_{wall}$, respectively, the surfaces for each patch (inlet, supra-aortic vessels, descending aorta, and wall), $A_{i}$ the area of the i outlet patch, N the total number of aortic outlets, n the normal unit vector, $u_{inlet}(t)$, $u_{out}^{ref}\left(t\right)$ are the time-varying values of the waveform inlet and outlet velocities, Figure 2, $p_{desc}\left(t\right)$ the time varying value of the waveform outlet pressure, Figure 3, and T the time period of the entire simulation.

### 2.2. Parameters Post-Processing

Once the governing Navier–Stokes equations are solved and the velocity and pressure fields are obtained, several derived hemodynamic quantities can be computed. These metrics offer additional insights into the mechanical environment experienced by the fluid and surrounding surfaces. To characterize the biomechanical environment within the thoracic aortic arch, four key hemodynamic quantities were computed in post-processing: WSS, OSI, DF, and TVR. Such parameters are widely used in biomedical-oriented fluid dynamics and are reported in [18].

#### 2.2.1. Wall Shear Stress (WSS)

WSS quantifies the tangential viscous force per unit area that the fluid exerts on the wall. It refers to the tangential force exerted by blood flow on the endothelial surface of the vessel wall; in the aorta, alterations in WSS play a crucial role in modulating endothelial cell function, contributing to processes such as inflammation, endothelial dysfunction, and vascular remodeling, which are central to the development of aortic pathologies [19,20,21]. The definition of WSS follows:

(6) $$WSS=\mu {\left(\frac{\partial u_{t}}{\partial n}\right)}_{wall}$$

with $u_{t}$ is the tangential component of the velocity at the wall, n the normal unit vector, and μ the dynamic viscosity. Regions of abnormally low or oscillatory WSS have been linked to the development of vascular pathologies such as atherosclerosis and aneurysms.

#### 2.2.2. Oscillatory Shear Index (OSI)

OSI quantifies the directional fluctuations of wall shear stress during the cardiac cycle and serves as a hallmark of disturbed flow. High OSI values are associated with disturbed or recirculating flow patterns, which are often observed in regions susceptible to vascular or disease [22]. In the aorta, elevated OSI has been linked—alongside low WSS—to regions predisposed to aneurysmal dilation and atherosclerotic plaque formation [23,24]. The OSI measures the degree of directional change in the WSS over a pulsatile cycle:

(7) $$OSI=\frac{1}{2}\left(1-\frac{\left|\int_{0}^{T}WSS\left(t\right)dt\right|}{\int_{0}^{T}WSS\left(t\right)|dt}\right)$$

where T is the duration of the cardiac or pulsatile cycle. OSI is a dimensionless quantity ranging from 0 (purely unidirectional flow) to 0.5 (oscillatory flow with zero net direction).

#### 2.2.3. Drag Forces (DF)

DF are the displacement forces exerted by pulsatile blood flow on arterial walls and stent-grafts; these forces play a critical role in graft migration and endoleak. CFD studies have demonstrated that DF magnitude and orientation vary across Ishimaru zones and are significantly influenced by factors such as the angulation and tortuosity of the proximal landing zone, graft length, diameter, deployment position, and patient-specific hemodynamics—with sideways and upward components contributing differently depending on the anatomical zone [13]. The local drag force represents the total force exerted by the fluid on a surface due to both pressure and viscous stresses:

(8) $$F_{D}=\int_{S}(-pn+\mu (\nabla u+{\left(\nabla u\right)}^{T})\cdot n)dS$$

The resulting force and its projection along a specified axis (e.g., the streamwise direction) can be used to characterize flow resistance or loading on surfaces such as medical devices, arterial walls, or prostheses [25]. Due to the negligible contribution of the viscous term, the DF is computed as follows:

(9) $$F_{D}=\int_{S}(-pn)dS$$

with S the surface area of the region.

#### 2.2.4. Turbulent Viscosity Ratio (TVR)

Quantifies the relative importance of turbulent momentum transport in vascular flow simulations. In aortic aneurysm models, elevated TVR values highlight regions of recirculation and transitional flow that can amplify hemodynamic disturbances. Excessive TVR in the aneurysm sac and proximal neck may coincide with increased risk of stent graft instability and endothelial stress abnormalities [26,27].

(10) $$TVR=\frac{\nu_{t}}{\nu }$$

where $\nu_{t}$ is the turbulent viscosity and ν the kinematic viscosity. In simulations that involve turbulence modeling, TVR accounts for the intensity of the turbulent effects over the viscous ones. It is especially important in modeling transitional or turbulent flows, for instance in stenotic arteries or flow past complex geometries [28].

### 2.3. Ishimaru Classification

The Ishimaru classification provides a standardized geometric framework for dividing the aortic arch into discrete zones, allowing the application of hemodynamic parameters and computational analysis techniques described earlier. This standardized segmentation enables both local and global evaluation of flow characteristics and facilitates the quantification of spatial variations in hemodynamic parameters along the aortic arch. This classification divides the aortic arch into anatomical zones to guide TEVAR:

- Zone 0: Includes the ascending aorta and the origin of the brachiocephalic artery.
- Zone 1: Extends from the brachiocephalic artery to the origin of the left common carotid artery.
- Zone 2: Extends from the left common carotid artery to the origin of the left subclavian artery.
- Zone 3: Located distal to the origin of the left subclavian artery, in the proximal descending thoracic aorta [29].

In cases of ASA, the standard Ishimaru classification has been adapted by Tinelli et al. [15] to account for the altered vascular anatomy:

- Zone 0: Includes the ascending aorta and the origin of both common carotid arteries, and the bicarotid trunk.
- Zone 1: Encompasses the segment of the aortic arch that includes the origin of the non-aberrant subclavian artery.
- Zone 2: Refers to the segment of the aortic arch that includes only the origin of the ASA.
- Zone 3: Located distal to the ASA origin, within the proximal descending thoracic aorta.

**Figure 4:**
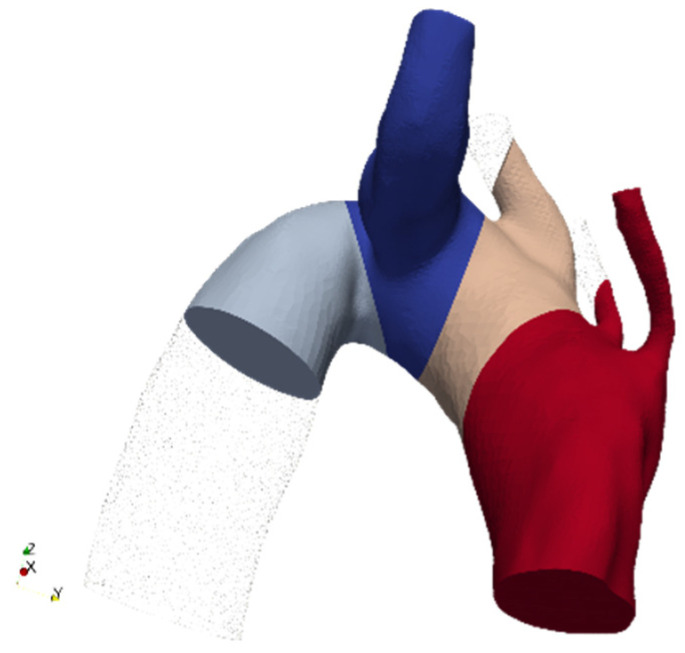
Ishimaru partitions example in the case of ASA (Aberrant Subclavian Artery).

Ishimaru’s zones’ visual representation is shown in Figure 4, in the case of ASA. To account for any initial transient phenomena, three cardiac cycles have been simulated, and results are computed solely for the last one, so to exclude numerical noise due to simulation initialization.

The four scalar or vector fields of interest in Ishimaru’s zones are derived from the velocity and pressure data obtained from CFD simulations and were analyzed using a two-level averaging procedure to reduce local variability and enable regional comparison. Each generic quantity ϕ was first averaged over the duration of a full cardiac cycle T, yielding a time-averaged distribution:

(11) $$<\phi >\left(x\right)=\frac{1}{T}\int_{0}^{T}\left|\phi (x,t)\right|dt$$

This step captures the cumulative mechanical stimulus over time, smoothing out transient oscillations while preserving relevant flow features. To enable anatomically consistent regional analysis, the aortic arch was partitioned according to the Ishimaru classification, which defines standardized cross-sectional planes along the curvature of the arch. For each Ishimaru zone, spatial averaging was performed over the local surface, for wall-based metrics like WSS, OSI, or volume (for volumetric fields like TVR), yielding a representative scalar for that region:

(12) $$\bar{\phi }=\frac{1}{\left|S\right|}\int_{S}<\phi (x)>dA$$

This double-averaging strategy in time and space allows for overall quantification and direct comparison between regions of the arch (e.g., ascending aorta, arch curvature, descending aorta), and supports correlation with anatomical features, pathological locations. It should be underlined that, in this study, averaged quantities and their standard deviation refer to the spatial and temporal variability of the hemodynamic quantities and have no statistical meaning, given the small population size. Average and standard deviation quantities are computed using the built-in temporal statistics function available with Paraview [30]. For any further information please refer to the Paraview guide. Lastly, average quantities for pathologic and healthy anatomies over each Ishimaru region are reported in a tabular form. Such comparison is performed by averaging numeric values of each hemodynamic scalar and field over each section over each patient.

## 3. Results

### 3.1. Patients’ Selection

This study includes six patients who were either treated or evaluated for treatment at our center. Five patients presented with the classical variant of a right ASA arising from a left-sided aortic arch, while one patient had the rarer form of a left ASA originating from a right-sided aortic arch (M6). The dataset description is summarized in Table 1 for healthy anatomies and in Table 2 for pathological ones.

Two patients had no associated aortic pathology, whereas the remaining four presented with acute aortic syndromes—one with a ruptured aortic arch aneurysm involving the KD (M1) and three with type A aortic dissections (M3–5). Among the six patients, all but one had a KD at the origin of the ASA, and two patients exhibited a bicarotid trunk.

### 3.2. WSS and OSI Analysis

**Figure 5:**
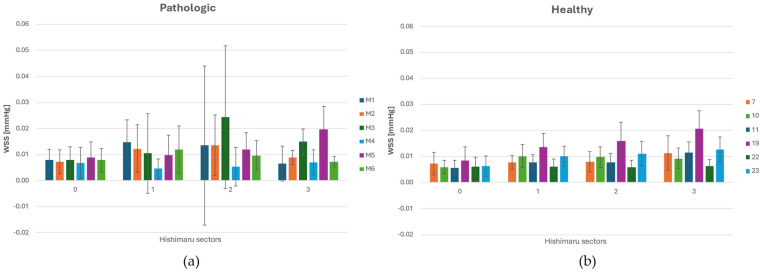
(**a**) WSS (Wall Shear Stress) distribution across each sector of the Ishimaru’s classification and spatial deviation for ASA vessels. (**b**) WSS distribution across each sector of the Ishimaru’s classification and spatial deviation for non-ASA vessels.

As can be seen in the histograms in Figure 5a,b, in zone 0, WSS levels were at the same magnitude and consistent across all anatomical configurations, consistent with accelerated flow in the ascending aorta. These findings reflect the proximity to the left ventricular outflow tract, where pulsatile inflow generates strong, forward-directed shear forces. Moving to zone 1, a progressive increase in WSS was observed, more accentuated in ASA cases. This rise is attributable to the beginning of flow deceleration and redistribution near the supra-aortic branches. In the presence of ASA, altered branching may contribute to early momentum loss and localized flow separation, increasing WSS magnitude.

The highest in value and fluctuating WSS values were consistently found in zone 2, particularly in geometries with an ASA. In this region, the presence of the ASA leads to complex flow interactions, including low-velocity recirculation zones. In zone 3, WSS decreases slightly compared to zone 2. In ASA cases, the distal arch continued to exhibit high WSS, due to persistent helical flow and skewed velocity profiles resulting from upstream disturbance. Normal geometries tend to recover higher WSS values more rapidly in this region. These results highlight that WSS is spatially heterogeneous along the aortic arch, with a significant rise in zones 2 and 3 in the presence of ASA.

**Figure 6:**
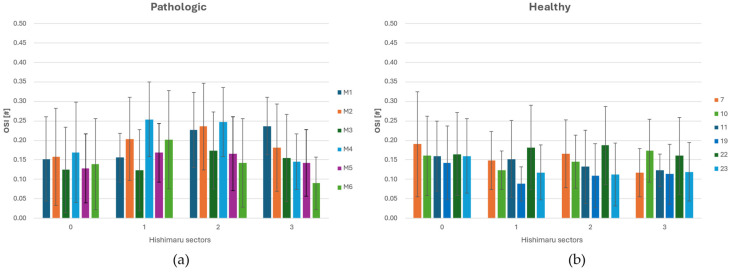
(**a**) OSI (Oscillatory Shear Index) distribution across each sector of Ishimaru’s classification and spatial deviation for ASA vessels. (**b**) OSI distribution across each sector of Ishimaru’s classification and standard deviation for non-ASA vessels.

Looking at the histograms in Figure 6a,b, across all configurations, OSI was lowest in zone 0, reflecting the straight geometry and stable flow regime of the proximal ascending aorta. Minimal differences were observed between normal and ASA cases in this region, consistent with the fact that upstream flow is unaffected by arch anomalies. In zone 1, a moderate rise in OSI was observed, more pronounced in ASA anatomies. This increase corresponds to the initial curvature of the arch and the influence of supra-aortic branch outflows, which begin to introduce secondary flow structures and WSS oscillations. Zone 2 exhibited the highest OSI values, particularly in the presence of an ASA. The complex vessel geometry and altered branch take-off generate strong multidirectional shear, leading to significantly elevated OSI magnitudes. In zone 3, OSI values decreased again while remaining higher in ASA cases than in the healthy ones. This residual elevation reflects the downstream propagation of helical or asymmetric flow patterns initiated in the arch. Overall, the sectorial OSI analysis highlights the regional sensitivity of zones 2 and 3 to anatomical variations, with the former zone emerging as the critical one where morphological anomalies translate into altered biomechanical stimuli on the vascular wall.

### 3.3. DF Analysis

**Figure 7:**
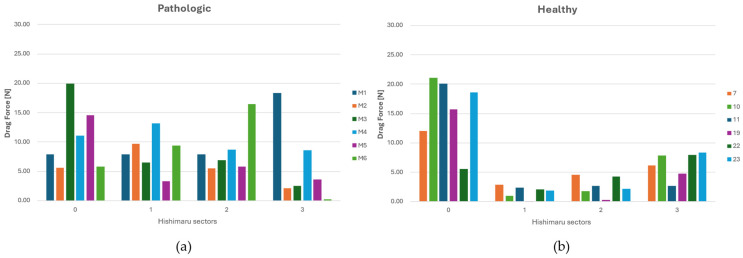
(**a**) DF (Drag Force) distribution across each sector of Ishimaru’s classification for ASA vessels. (**b**) DF distribution across each sector of Ishimaru’s classification for non-ASA vessels.

For the Ishimaru sector, DF magnitudes were extracted and compared across individuals. The aberrant cohort demonstrated marked inter-subject variability, with multiple sectors—particularly in zones 2 and 3—exhibiting consistently elevated drag forces, as shown in Figure 7a. This pattern suggests localized alterations in flow dynamics, attributable to perturbed geometry and asymmetric branching associated with the aberrant vessel.

Conversely, the control anatomy yielded lower and more homogeneous DF profiles across all zones, consistent with physiologically streamlined flow, as shown in Figure 7b. Since each patient exhibits a specific DF pattern closely related to their individual aortic arch geometry, the data from different cases cannot be aggregated. However, in pathological cases, a tendency can be observed: in zone 2, the DF typically shows a posterosuperior orientation, while in zone 1, the direction of the drag force is more variable, displaying either anterosuperior or posterosuperior trajectories.

### 3.4. TVR Analysis

In zone 0, TVR values were low across all anatomical configurations, indicating stable inflow from the ascending aorta. This is consistent with the high WSS and low OSI observed in this region, suggesting a low-turbulence flow.

**Figure 8:**
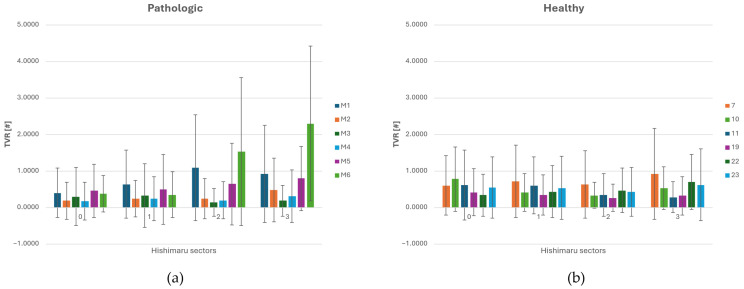
(**a**) TVR (Turbulent Viscosity Ratio) distribution across each sector of Ishimaru’s classification and standard deviation for ASA vessels. (**b**) TVR distribution across each sector of Ishimaru’s classification and spatial deviation for non-ASA vessels.

Although the rise was not remarkable in value, it suggests early signs of flow instability beginning to emerge. In zone 2, TVR values increased markedly, especially in the presence of ASA, as shown in Figure 8a.

A clear increase in the TVR’s average value and spatial oscillation is noted in zones 2 and 3 for aneurysmatic (M1) and strongly angulated geometries (M6), downstream of the ASA. Moreover, such cases also presented increasing spatial oscillations proceeding from zone 0 to zone 3. Throughout the other cases, TVR’s average values and spatial oscillations remained substantially stable. Interestingly, TVR values in zone 0 and zone 1 tend to be lower in pathological cases compared to the healthy ones, as shown in Figure 8b. This phenomenon can be attributed to the presence of the carotid arteries in these regions. Due to their smaller size and lower flow rates, the carotids exert a reduced influence on the dynamics of the main aortic flow, resulting in lower vorticity transfer in the proximal aortic arch. The interaction between curved geometry and altered branching patterns appears to enhance vorticity and local instabilities, giving rise to elevated TVR levels. Zones 2 and 3 align spatially with the highest OSI and WSS regions, supporting the interpretation that flow in this area is both disturbed and energetically disordered. The higher TVR in zone 3 in ASA cases suggests that flow perturbations persist downstream of the aberrant branch, due to continued helical structures or skewed velocity profiles carried into the descending thoracic aorta. Overall, the spatial analysis of TVR reveals that ASA anatomy significantly increases local flow complexity in zones 2 and 3.

### 3.5. Sector-Wise Analysis

In Table 3, average values of each parameter are reported along every Ishimaru zone. OSI was consistently higher in the pathological cases, indicating greater temporal variability of shear stress, and thus a more disturbed and oscillatory flow environment [16]. Similarly, WSS tended to be higher and more oscillating in the pathological anatomy in most regions, suggesting increased mechanical stress on the vessel walls [25]. Interestingly, Drag Force exhibited regional variability: it was markedly higher in the healthy anatomy in zone 0, while in zones 1 and 2, it was elevated in the pathological case [26]. TVR also shows marked differences, with lower values in zone 0 and higher values in zone 3 in the pathologic group, suggesting it may capture cumulative effects of disturbed flow.

### 3.6. Representative Cases

**Figure 9:**
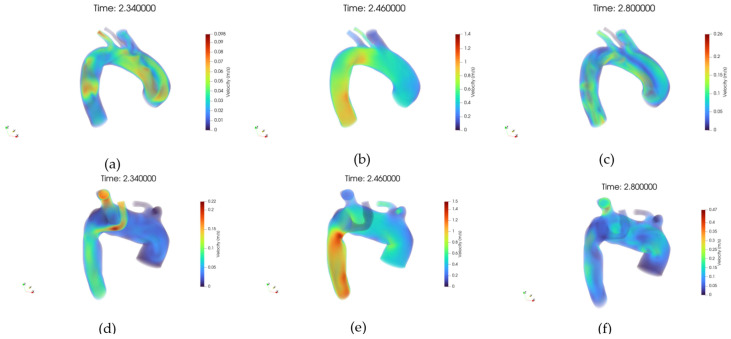
(**a**) Volume rendering of the velocity (U) field at early systole, case 10. (**b**) Volume rendering of the velocity field at peak systole, case 10. (**c**) Volume rendering of the velocity field at diastole, case 10. (**d**) Volume rendering of the velocity field at early systole, case M3. (**e**) Volume rendering of the velocity field at peak systole, case M3. (**f**) Volume rendering of the velocity field at diastole, case M3.

Figure 9a–f presents the three-dimensional velocity field visualized via volume rendering, respectively, for the healthy and pathological anatomies, alongside the inlet velocity imposed, Figure 9b.

In the healthy case, the velocity field is characterized by a coherent and streamlined flow pattern throughout the aortic arch. High-velocity regions are concentrated in the center of the lumen, forming a well-defined core flow, while near-wall regions exhibit gradual velocity decay, consistent with physiological flow behavior.

No signs of large-scale flow separation or recirculation zones are observed, and the symmetry between anterior and posterior views further supports the presence of stable hemodynamics. In contrast, the pathological anatomy reveals markedly altered velocity distributions.

The volume rendering highlights irregular flow structures, with regions of reduced velocity extending across wider areas of the lumen. Flow asymmetry is evident, particularly around the ASA, where disturbed patterns and potential vortex formation are suggested by the diffuse and disorganized velocity gradients. These observations align with the presence of anatomical anomalies that disrupt the natural flow path, leading to locally reduced shear and potentially increasing the risk for vascular remodeling or thrombogenic conditions.

**Figure 10:**
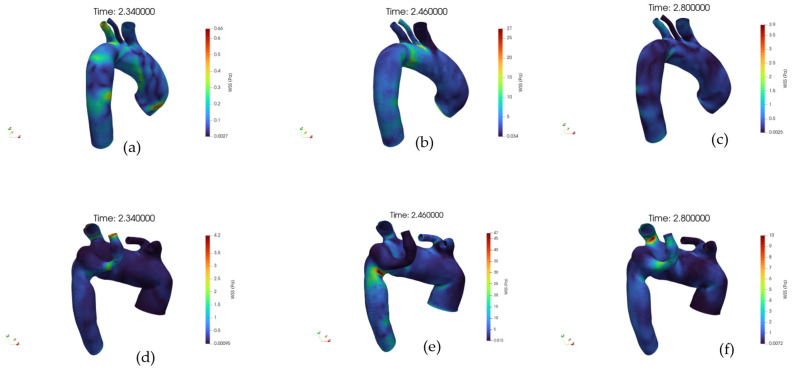
(**a**) Volume rendering of the Wall Shear Stress (WSS) field at early systole, case 10. (**b**) Volume rendering of the WSS field at peak systole, case 10. (**c**) Volume rendering of the WSS field at diastole, case 10. (**d**) Volume rendering of the WSS field at early systole, case M3. (**e**) Volume rendering of the WSS field at peak systole, case M3. (**f**) Volume rendering of the WSS field at diastole, case M3.

Figure 10a–f shows the WSS distribution at the wall, respectively, for the healthy and pathological anatomies. In a healthy artery, WSS shows a spatially homogeneous distribution that increases consistently from early systole to peak systole, followed by a marked reduction during diastole.

The high-WSS regions during systole are aligned with the main flow direction, suggesting a stable shear environment. Conversely, the pathological artery exhibits a markedly altered WSS pattern. At early systole, low WSS zones are already apparent in branching or geometrically perturbed regions.

During peak systole, although WSS intensifies globally, the distribution remains heterogeneous, with sharp spatial gradients and isolated hotspots of elevated shear adjacent to extended low-shear regions. In diastole, the WSS dramatically decreases, yet large areas persist with near-zero shear. The pathological anatomy appears to induce complex, non-uniform WSS distributions across the cycle, in stark contrast with the controlled, uniform shear environment of the healthy vessel.

**Figure 11:**
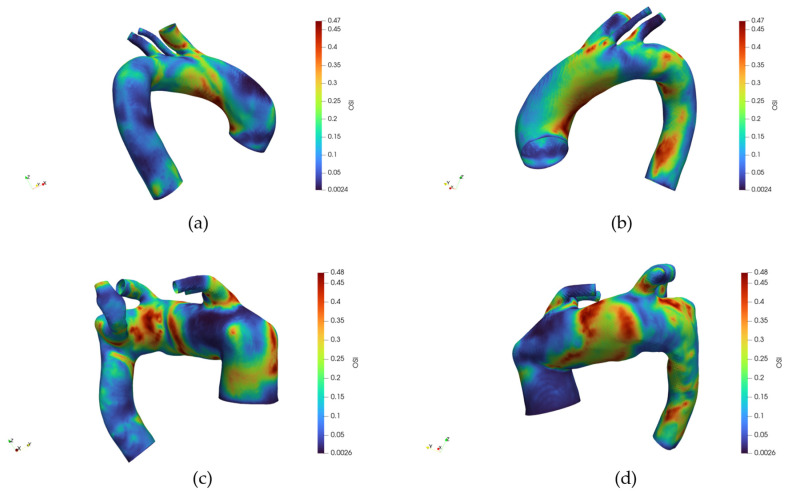
(**a**) Distribution of the Oscillatory Shear Index (OSI) for the healthy reference case, frontal view. (**b**) Distribution of the OSI for the healthy reference case, back view. (**c**) Distribution of the OSI for the pathologic reference case, frontal view. (**d**) Distribution of the OSI for the pathologic reference case, back view. The different color belong to different value of the Drag Force in Newton.

Figure 11 illustrates the spatial distribution of the OSI across the aortic surface, respectively, for healthy and pathological anatomy, each displayed from anterior and posterior perspectives. In a healthy subject, OSI values remain consistently low across most of the vessel wall, indicating a predominance of unidirectional shear stress.

Slightly elevated OSI regions are localized near the branch ostia, particularly at the origins of the supra-aortic vessels, yet remain confined and limited in magnitude. The posterior wall demonstrates a similarly low OSI, with no significant focal elevations. Conversely, the pathological anatomy displays a markedly different OSI pattern. Elevated OSI regions are more extensive and distributed asymmetrically, especially around the inner curvature of the aortic arch and along the posterior wall. The retrograde view reveals prominent high-OSI patches in proximity to the origin of the aberrant subclavian artery and adjacent areas, suggesting disturbed and oscillatory flow patterns.

**Figure 12:**
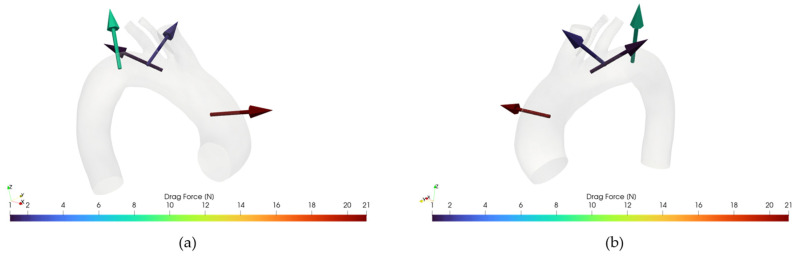
(**a**): Drag Force for case 10, retro view. (**b**) Drag Force for case 10, frontal view.

**Figure 13:**
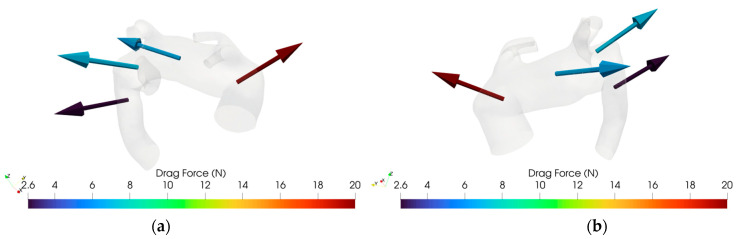
(**a**): Drag Force for case M3, retro view. (**b**) Drag Force for case M3, frontal view.

Figure 12 and Figure 13 illustrate an example of DF vector and magnitude analysis across the different zones of the aortic arch for reference healthy and pathologic cases.

**Figure 14:**
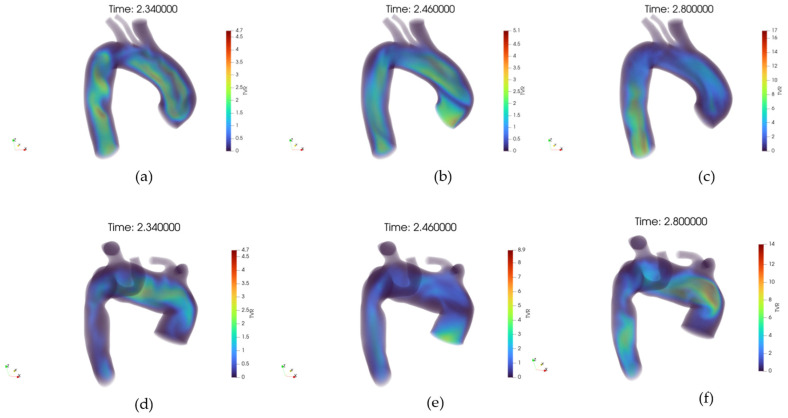
(**a**) Volume rendering of the Turbulent Viscosity Ratio (TVR) field at early systole, case 10. (**b**) Volume rendering of the TVR field at peak systole, case 10. (**c**) Volume rendering of the TVR field at diastole, case 10. (**d**) Volume rendering of the TVR field at early systole, case M3. (**e**) Volume rendering of the TVR field at peak systole, case M3. (**f**) Volume rendering of the TVR field at diastole, case M3.

In Figure 14, the volume renderings of TVR for early systole, peak systole, and diastole for cases 10 and M3 are reported, respectively.

A visual comparison reveals a marked increase in TVR in the ASA subject compared to the healthy control across all phases. During the pre-systolic phase, the reflux volume is limited in both cases, but differences are already apparent: in the healthy subject, the TVR is confined to proximal and central segments, whereas in the ASA case, it extends toward distal regions of the aortic arch. At the systolic peak, the healthy subject exhibits a substantial reduction in TVR, reflecting efficient pulsatile emptying of the arterial lumen. Conversely, the ASA subject shows persistent and significant reflux volumes distributed along the entire arch, including the ASA. During diastole, the healthy subject exhibits a modest increase in TVR. However, in the ASA subject, TVR reaches its highest observed volume, with a broad spatial extension involving not only the proximal brachiocephalic trunk but also distal branches, including the aberrant subclavian artery.

## 4. Discussion

This study investigated the anatomical influence of the ASA on WSS, OSI, DF, and TVR hemodynamic quantities. The authors exploited CFD to evaluate the spatial, time-averaged behavior and oscillations of such parameters. Given the relatively small dataset, the results cannot be statistically analyzed. However, the technical feasibility and reliability have been assessed as a first step towards a statistically relevant future study.

Summarizing what has been shown in Section 3, we can infer that in zone 0, corresponding to the ascending aorta, no significant hemodynamic differences were observed between pathological and healthy groups, except for slightly higher DF magnitudes in healthy anatomies. Moreover, the WSS average value is greater in magnitude for pathological cases, as shown in Table 3 and in the histograms (Figure 5, Figure 6, Figure 7 and Figure 8). This peculiarity suggests a more disturbed flow in such regions for pathologic cases affected by downflow arc abnormalities. This region is directly influenced by the left ventricular outflow, resulting in streamlined, unidirectional flow patterns. These results align with previous CFD studies on the ascending aorta, which describe this segment as exhibiting relatively stable shear environments unless altered by root dilatation or valve dysfunction [21,31]. Therefore, the lack of marked differences in zone 0 does not support a hemodynamic explanation for the increased incidence of type A dissections in patients with KD, suggesting that other mechanisms—such as underlying histopathological alterations [7] may play a more prominent role in proximal aortic complications. Zones 1 through 3 showed pronounced hemodynamic disturbances in patients with ASA, particularly at the ostium of the ASA in zone 2. Notably, these regions demonstrated a marked increase in WSS and OSI, as well as significantly elevated drag force magnitudes and turbulent viscosity ratios (TVR). This pattern reflects a mechanically complex flow environment characterized by oscillatory shear, fluctuating load vectors, and intensified turbulence—all of which are known to promote endothelial dysfunction, pro-inflammatory gene expression, and extracellular matrix degradation [32,33]. Collectively, these features indicate a state of chronic wall fatigue and elevated biomechanical stress, creating an environment conducive to pathological remodeling. These findings support the hypothesis that the aortic arch in ASA patients represents a hemodynamic hotspot, where the confluence of altered geometry and disturbed flow can contribute to aneurysmal degeneration at the ASA origin and the increased risk of dissection or rupture in these patients.

Particularly interesting are patients M1 and M6, who exhibited the highest DF and TVR values in zones 2 and 3. Patient M1 presented with a large distal arch aneurysm, while patient M6 had a right-sided aortic arch with pronounced angulation—both anatomical factors likely exacerbating flow disruption and wall stress. These cases highlight the individual variability in hemodynamic burden, reinforcing the value of patient-specific analysis. Notably, our findings also carry practical implications for endovascular repair. Drag force vectors were smaller and more axially aligned in zone 1, suggesting that this region may represent a safer and more stable proximal landing zone for TEVAR. Conversely, zone 2—particularly near the ASA ostium—showed larger, more oblique drag vectors, potentially increasing stent graft stress and the risk of migration or endoleak. These observations are consistent with prior work by Domanin et al. [13], who emphasized the role of flow-induced forces in device stability. Thus, CFD-based DF mapping may serve as a valuable adjunct to anatomical assessment in guiding stent deployment strategies in complex arch anatomies. Moreover, our analysis demonstrates that ASA is associated with significant hemodynamic disturbances in the aortic arch, particularly in zones 2 and 3. While zone 0, corresponding to the ascending aorta, exhibited comparable WSS and OSI values in both groups, zones 1 through 3 showed pronounced perturbations in pathological cases. In ASA patients, we observed marked increases in WSS, OSI, DF, and TVR, especially at the ASA ostium in zone 2. These findings are consistent with prior CFD studies identifying the distal arch as a hemodynamic “hotspot” prone to recirculation zones, oscillatory shear, and complex vertical structures [19].

This study demonstrates how CFD can provide critical insight into the pathogenesis of aortic disease. By quantifying flow-derived mechanical stimuli that are otherwise inaccessible through conventional imaging, CFD offers a mechanistic interpretation of how anatomical features may suggest degeneration for certain regions of the aorta. Similar approaches have been successfully applied in other contexts, such as in the study of bicuspid aortic valve-associated aortopathy [34] and aortic aneurysm progression [35,36], confirming that local hemodynamics play a key role in disease evolution. Our findings extend this evidence base to rare arch anomalies such as ASA and KD, proposing a plausible flow-mediated mechanism underlying their clinical behavior. Beyond pathophysiological insight, CFD also offers practical value in preoperative planning for TEVAR. In particular, quantifying drag forces along the aortic wall can help identify optimal landing zones, improving stent graft stability and reducing the risk of migration or endoleak [13]. Our study revealed a tendency toward more favorable DF values in zone 1, where drag vectors were smaller and more axially aligned. Conversely, in zone 2—especially near the ASA ostium—drag forces were often larger and more obliquely oriented, potentially increasing mechanical stress on the endograft. These findings suggest that DF mapping via CFD may be a valuable addition to standard anatomical assessment, particularly in anatomically complex settings such as in the presence of ASA.

## 5. Conclusions

This study provides a comprehensive hemodynamic analysis of patients with ASA anatomy, using CFD simulations to assess the distribution of interest hemodynamic quantities along the aortic arch. By leveraging sector-based quantification through the Ishimaru classification, we demonstrated consistent patterns of altered force distribution in ASA subjects when compared to a control case with normal anatomy. These differences suggest that the presence of an ASA may contribute to localized hemodynamic stress, potentially predisposing affected regions to vascular remodelling or pathological progression. However, this study is intended to be performed following a statistical approach on a larger cohort in future, considering the rarity of such congenital anomaly.

ASA alters aortic arch hemodynamics: patients with an ASA show distinct hemodynamic quantity distributions compared to those with normal anatomy. Unfavorable landing zones in ASA patients:

- Zone 2: Typically displays high drag force and significant variability, likely due to disturbed flow from the aberrant vessel. Hence, it should be avoided for stent anchoring.
- Zone 3: May experience steep force gradients, possibly related to geometric transitions before the ASA origin. Hence, represents a less stable landing site.

Regions with high OSI suggest a more oscillating WSS, in magnitude and direction. Regions with greater TVR suggest unstable flow patterns and larger, low velocity, vorticose zones, especially in the aneurysmatic case (M1) and angulated (M5 and M6) anatomies. CFD is a powerful non-invasive tool for assessing powerful insights into hemodynamics, with lower costs compared to other diagnostic techniques. Moreover, by gathering ordered and organized CFD data on such anatomical anomalies in a unique dataset, this study is presented as a starting point towards a more detailed, personalized diagnosis.

WSS is found to be highly oscillating in ASA cases, contrary to healthy ones, indicating also greater OSI values, especially in zones 2 and 3, where the ASA lies.

ASA anatomy significantly alters aortic arch hemodynamics, with zones 2 and 3 emerging as focal regions of elevated shear stress, oscillatory flow, and drag forces. These disturbances create a pro-remodeling environment that may underlie the increased risk of aneurysm progression, rupture, and dissection in ASA patients. Importantly, CFD analysis not only elucidates these mechanisms but also offers clinical utility by identifying favorable landing zones and anticipating endograft forces during TEVAR. Incorporating patient-specific CFD into the evaluation of ASA—particularly in asymptomatic cases—could enhance surveillance strategies and guide personalized treatment planning. Larger prospective studies are required to validate these findings and translate CFD into routine clinical practice.

**Figure 15:**
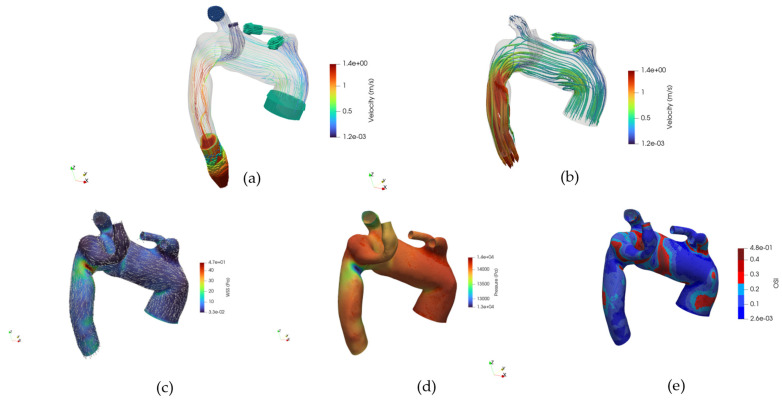
(**a**) Navigable velocity streamlines and patch glyphs, (**b**) Navigable velocity streamlines glyphs, (**c**) Navigable WSS glyphs and distribution map, (**d**) Navigable pressure field at the wall, (**e**) Navigable OSI.

Our findings underscore the importance of incorporating detailed anatomical variations into hemodynamic assessments and highlight the value of CFD as a non-invasive tool to investigate patient-specific vascular risks. Moreover, with such a study, a complete dataset for CFD simulation of the ASA is provided. CFD-ready geometries are brought to the disposal of the scientific community, together with boundary conditions and hemodynamic fields. The dataset enables the visualization and navigation of interest hemodynamic fields to extensively investigate such abnormalities in the thoracic aorta’s anatomy. The following post-processing is gathered into a Paraview state and shown in Figure 15:

- Internal velocity streamlines and velocity glyphs at the patch
- Velocity streamlines’ glyph
- WSS distribution map and direction glyph on the wall
- Pressure at the wall
- OSI distribution map
- Internal fluid’s velocity
- Internal fluid’s pressure

Hemodynamic fields are collected at the same time-steps considered in this work: early systole, systolic peak, and diastole.

This study aims to employ CFD as a complementary tool to study vascular disease pathogenesis and to assess a patient’s hemodynamic status in the context of potential surgical intervention. The boundary conditions used in the simulations are not patient-specific; this choice was made intentionally to isolate the effect of ASA anatomy on the resulting flow fields under identical hemodynamic conditions. However, studies to assess patients’ conditions and state remain impactful in merely clinical terms. Therefore, future studies may address this by simulating the same anatomical configurations with boundary conditions derived directly from patient-specific data and following any eventual stent migration from post-operative observations. Furthermore, specialized engineering personnel reconstructed vascular geometries. Although this ensures high-quality models, it may represent a constraint in routine clinical settings. To address this issue, future work will focus on developing CFD simulations based on geometries automatically reconstructed and patched using artificial intelligence, thereby facilitating analysis by clinicians. A similar approach has been demonstrated in a previous paper [37].

In future perspectives, this research may open several avenues for extension. First, enriching the study cohort with additional uncomplicated ASA patients would allow for a more precise definition of the early geometric and hemodynamic patterns that may predispose to pathology. The statistical approach is for now left to future studies, bearing in mind the rarity of such congenital anomaly, which makes the data collection process highly time demanding. Such data could improve our understanding of disease initiation, rather than just its complications. Second, we plan to simulate various endovascular treatment scenarios, assessing how different stent graft configurations and landing zones affect hemodynamic parameters post-deployment. This could pave the way for personalized treatment planning, optimizing both anatomical fit and biomechanical compatibility.

The data used and generated in this study are available on Zenodo, within a continuously expanding database dedicated to clinical cases with ASA. This initiative aims to support the scientific community in the modeling, analysis, and understanding of hemodynamics in rare anatomical conditions. The dataset can be accessed and downloaded at https://doi.org/10.5281/zenodo.15835201 (accessed on 7 October 2025) under the CC BY 4.0 Attribution 4.0 International License, which requires researchers using this dataset to cite this paper as the primary reference. https://creativecommons.org/licenses/by/4.0/ (accessed on 7 October 2025).

## Figures and Tables

**Table 1:** Patient 1: Healthy reference population description.

#	Gender, Age	Ethnic Group
7	M, 90	Caucasian
10	M, 79	Caucasian
11	M, 90	Caucasian
19	M, 78	Caucasian
22	M, 76	Caucasian
23	M, 61	Caucasian

**Table 2:** ASA patients’ pathological and anatomical description.

#	Gender, Age	Ethnic Group	ASA and Associated Aortic Lesion	Right or Left-Sided Arch	KD	Retro-Esophageal ASA	Bicarotid Trunk
M1	F, 74	Caucasian	Ruptured arch aneurysm (zone 2 and 3) + ARSA	Left	Yes	Yes	No
M2	M, 80	Caucasian	Symptomatic ARSA	Left	Yes	Yes	No
M3	F, 73	Caucasian	TAAD (Type A Aortic Dissection) + ARSA	Left	Yes	Yes	Yes
M4	M, 76	Caucasian	TAAD + ARSA	Left	Yes	Yes	No
M5	M, 34	Caucasian	TAAD + ARSA	Left	No	Yes	Yes
M6	F, 80	Caucasian	Asymptomatic ALSA	Right	Yes	Yes	No

**Table 3:** Average values across all patients in the Ishimaru sector of the interest quantities.

Ishimaru Region	0		1		2		3	
	Pathologic	Healthy	Pathologic	Healthy	Pathologic	Healthy	Pathologic	Healthy
DF [N]	10.83	15.51	8.32	1.72	6.78	2.61	6.46	6.29
OSI [#]	0.15	0.16	0.18	0.13	0.20	0.14	0.16	0.13
WSS [Pa]	1.03	0.89	1.41	1.24	1.74	1.29	1.43	1.59
TVR [#]	0.31	0.54	0.38	0.50	0.64	0.41	0.83	0.56

# stands for dimensionless number. N stands for Newton.

## Data Availability

The data presented in this study are available publicly at the Zenodo repository https://doi.org/10.5281/zenodo.15835201 (accessed on 7 October 2025).
